# Sustained Health Care–Associated Infection Reduction Among Electronic Hand Hygiene Monitoring System Clients

**DOI:** 10.2196/101905

**Published:** 2026-08-24

**Authors:** Gabrielle Genovesi

**Affiliations:** 1 BioVigil Technologies, LLC., 924 N. Main Street, Suite 2, Ann Arbor, MI, 48104, United States, 1 2489905711

**Keywords:** electronic hand hygiene monitoring, infection reduction, clinical outcomes, patient safety, hand hygiene compliance, CMS quality reporting, health care–associated infections, Centers for Medicare & Medicaid Services

## Abstract

This multifacility retrospective analysis of 25 hospitals using BioVigil Technologies’ electronic hand hygiene monitoring system demonstrated a mean of 46.95% (95% CI 35.5%‐58.4%) sustained reductions in health care–associated infections in 2024 compared to pre-implementation baselines, highlighting the clinical and operational value of automated hand hygiene monitoring at scale.

## Introduction

Health care–associated infections (HAIs) remain a challenge for health care facilities worldwide, contributing to patient morbidity, mortality, and substantial economic burden. In the United States, approximately 1 in 31 hospitalized patients acquires at least 1 HAI, at a cost of billions annually [1]. Despite longstanding evidence-based guidance, sustained improvements in infection rates have proven difficult across diverse health care settings.

Electronic hand hygiene monitoring (EHHM) systems offer a promising approach to enhancing adherence to infection prevention protocols while providing robust data for operational decision-making. By delivering real-time feedback to health care workers and tracking performance at the unit and facility level, these systems can identify and correct lapses in hand hygiene behavior, a primary contributor to HAIs [2]. Prior studies demonstrated that automated monitoring improves compliance and reduces infection rates, though reported effects vary by system design. A recent meta-analysis found EHHM increased compliance approximately 1.6-fold and was associated with markedly lower HAI rates, while trials of group-level dispenser-counting and proximity-based badge systems have shown more modest or inconsistent gains; long-term, multifacility analyses remain limited [3].

This analysis evaluates HAI performance among 25 BioVigil client facilities using publicly reported Centers for Medicare & Medicaid Services (CMS) data. Differing from group-level dispenser counters or proximity-based badges, BioVigil’s EHHM technology chemically verifies hand sanitization; confirms soap-and-water wash duration; and its color-coded badge provides real-time feedback through visual, vibrational, and audible cues, supporting cross-contamination prevention and individual-level accountability. By comparing pre- and post-implementation periods, we aim to quantify the clinical and economic impact of EHHM across diverse hospital types and sizes, providing insight into strategies for infection prevention improvement.

## Methods

### Study Design

Facilities were included if BioVigil’s EHHM was implemented in 2023 or earlier, CMS HAI data were complete for 2024 and the year preceding implementation, and CMS reporting was available at the individual facility level. These criteria yielded 25 facilities.

The facilities included 16 short-term acute care hospitals, 1 long-term acute care hospital, seven critical access hospitals, and one Veterans Affairs hospital, collectively comprising 4198 beds and 284 clinical units. CMS-reported HAIs for short-term acute care hospitals included catheter-associated urinary tract infections, central line-associated bloodstream infections, methicillin-resistant *Staphylococcus aureus*, surgical site infections, and *Clostridioides difficile* infections; long-term acute care, critical access, and Veterans Affairs hospitals reported catheter-associated urinary tract infections, central line-associated bloodstream infections, and *Clostridioides difficile* infections. Earliest full EHHM implementation occurred in 2016 (2 facilities) and most recently in 2023 (3 facilities).

This retrospective, observational analysis evaluated HAI outcomes aggregated at the hospital level. Percent change from baseline to 2024 was calculated for each facility and across the study population. Descriptive statistics, including mean, median, and 95% CIs, characterized sustained changes in infection counts. EHHM operational data from 2024 were also analyzed to quantify hand hygiene opportunities, real-time corrective reminders, and overall compliance rates. All data were publicly available CMS reports or deidentified operational records; no patient-level data were used.

### Ethical Considerations

This study utilized only deidentified, publicly available datasets from the CMS and summarized medical device usage counts. Because no individual-level or identifiable patient information was accessed or analyzed, this research did not constitute human subjects research, and institutional review board review was not required.

## Results

In 2024, BioVigil’s EHHM system captured 67,663,570 hand hygiene opportunities across all 25 facilities. During this period, 1,112,148 potential cross-contamination events were corrected in real time through badge-based reminders. This contributed to an average hand hygiene compliance rate of 91.25% across the study population.

Of the 25 facilities, 22 demonstrated reductions in total HAI counts compared to their pre-implementation baseline. Four facilities achieved net-zero HAIs in 2024, representing a 100% reduction. One facility exhibited no change, and 2 experienced minimal increases of 2 and 4 cases, respectively—both from unusually low baseline years that likely underrepresent each facility’s true pre-EHHM infection burden (Table 1).

**Table 1.** Facility characteristics and health care–associated infection (HAI) reductions in 2024.

Faculty name	Facility type	Approximate bed count (rounded to nearest 25)	BioVigil contract start year	HAI percent decrease in 2024
Hospital 1	Critical access	25	2023	100
Hospital 3	Critical access	25	2018	100
Hospital 10	Short-term acute care	50	2021	100
Hospital 15	Critical access	25	2021	100
Hospital 19	Short-term acute care	150	2023	82.35
Hospital 16	Short-term acute care	50	2021	75
Hospital 21	Short-term acute care	375	2021	71.43
Hospital 24	Veteran's Affairs	350	2023	66.67
Hospital 22	Short-term acute care	125	2022	64.71
Hospital 25	Short-term acute care	300	2014	64.06
Hospital 23	Short-term acute care	300	2018	55.71
Hospital 18	Long-term acute care	275	2020	54.55
Hospital 7	Critical access	25	2021	50
Hospital 20	Short-term acute care	75	2016	46.15
Hospital 8	Critical access	25	2021	44.44
Hospital 2	Short-term acute care	150	2021	39.13
Hospital 4	Short-term acute care	750	2020	37.33
Hospital 5	Short-term acute care	175	2017	24.32
Hospital 11	Short-term acute care	100	2021	20
Hospital 6	Short-term acute care	300	2020	17.46
Hospital 9	Short-term acute care	200	2021	13.04
Hospital 13	Short-term acute care	150	2021	11.11
Hospital 12	Critical access	25	2021	0
Hospital 17	Short-term acute care	460	2019	–13.79
Hospital 14	Critical access	25	2021	–50

**Figure 1.**
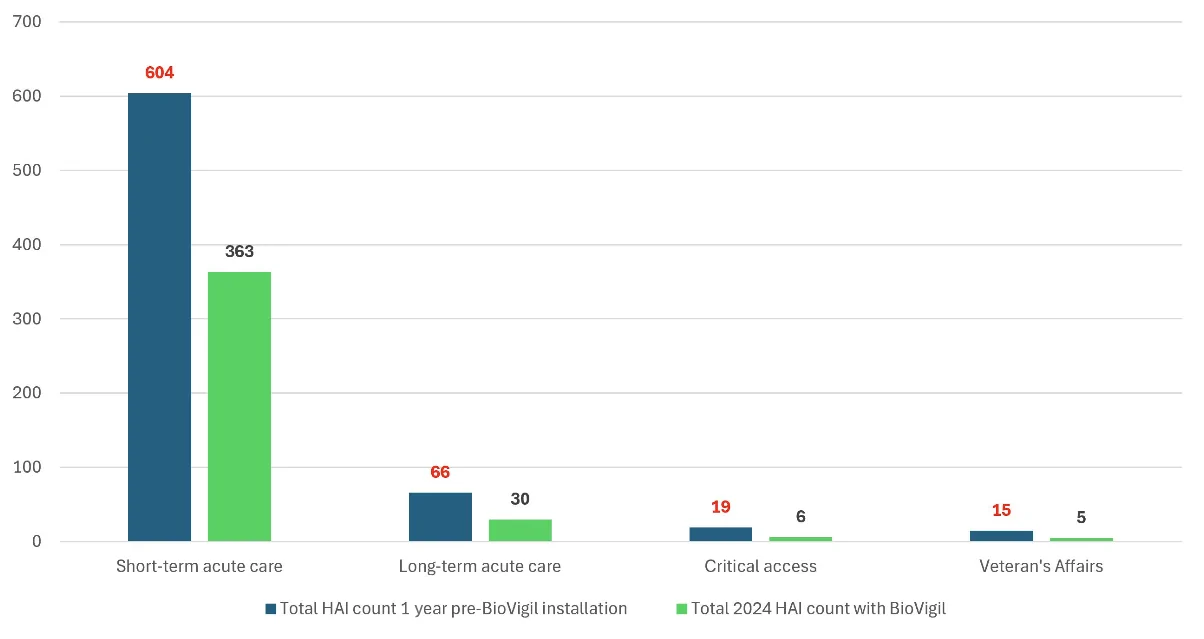
Health care–associated infection (HAI) annual counts pre–BioVigil electronic hand hygiene monitoring (EHHMS) installation versus the 2024 performance with EHHMS (by hospital type; 22 facilities).

Across all 25 facilities, the mean sustained HAI reduction was 46.95% (95% CI 35.5%‐58.4%), closely aligning with the median of 50% (IQR 30.83%-73.22%) (Figure 1). Among the 22 improving facilities, the mean reduction was 56.25% (95% CI 43.2%‐69.3%) with a median of 55.13% (IQR 37.78%-74.11%), collectively representing 300 fewer CMS-reported HAI cases in 2024. For context, national National Healthcare Safety Network data from over 38,000 US health care facilities showed year-over-year declines of only 2%‐11% across the 6 CMS-reportable HAI categories during the same period [4].

Financial and operational efficiencies were also observed, estimated using external benchmark averages. At a mean cost of $29,412 per HAI case [5], the observed reduction corresponds to approximately $8.82 million in avoided costs, excluding extended length of stay, readmissions, and CMS value-based purchasing penalties. Additionally, 16 of 25 facilities reported hand hygiene data to the Leapfrog Group; benchmark labor estimates suggest exclusive reliance on manual observation would have required approximately 136,620 staff hours and $5.74 million in labor costs in 2024 [6].

## Discussion

This analysis demonstrates that the large majority of BioVigil Technologies’ client facilities achieved meaningful, sustained reductions in HAIs in 2024. These findings extend prior evidence of EHHM’s effectiveness by documenting outcomes across a geographically and structurally diverse portfolio of 25 facilities over extended post-implementation periods. This is an analytical scope not well represented in existing literature, which has largely focused on single-site studies or short-term compliance metrics [3].

While prior work has established that automated monitoring improves adherence, this analysis suggests that sustained, high-level compliance translates into durable reductions in reported infection counts. This suggests EHHM may promote lasting behavioral change beyond attributable observation effects. From clinical and policy perspectives, these results reinforce the case for broader institutional investment in scalable, automated monitoring as a complement to existing infection prevention infrastructure.

This analysis has a few limitations to consider. First, as a retrospective, observational study without a randomized control group, causal attribution between EHHM implementation and HAI reductions cannot be definitively established, as additional infection prevention initiatives may have contributed. Second, baseline comparisons relied on a single pre-implementation year, which may not fully capture natural year-to-year variability, particularly in smaller facilities with low baseline counts. Third, the study population was limited to BioVigil clients that met the inclusion criteria, which may introduce selection bias and limit generalizability.

In conclusion, the majority of BioVigil client facilities experienced substantial HAI reductions in 2024 relative to pre-implementation baselines. This is regardless of system use duration. These outcomes suggest EHHM is an effective, sustainable, data-driven strategy to strengthen infection prevention performance, improve patient safety, and enhance health care efficiency.

## Abbreviations

CMS: Centers for Medicare & Medicaid Services
EHHM: electronic hand hygiene monitoring
HAI: health care–associated infection
